# Low doses of Bisphenol S affect post-translational modifications of sperm proteins in male mice

**DOI:** 10.1186/s12958-020-00596-x

**Published:** 2020-05-28

**Authors:** Hedvika Řimnáčová, Miriam Štiavnická, Jiří Moravec, Marouane Chemek, Yaroslav Kolinko, Olga García-Álvarez, Peter R. Mouton, Azalia Mariel Carranza Trejo, Tereza Fenclová, Nikola Eretová, Petr Hošek, Pavel Klein, Milena Králíčková, Jaroslav Petr, Jan Nevoral

**Affiliations:** 1 Biomedical Center in Pilsen, Faculty of Medicine in Pilsen, Charles University, alej Svobody 1655/76, Pilsen, Czech Republic; 2 LR11ES41: Génétique, Biodiversité et Valorisation des Bioressources, Institut de Biotechnologie, Université de Monastir, 5000 Monastir, Tunisia; 3 Department of Histology and Embryology, Faculty of Medicine in Pilsen, Charles University, Pilsen, Czech Republic; 4 SaBio IREC (CSIC-UCLM- JCCM), Albacete, Spain; 5 SRC Biosciences & University of South Florida, Tampa, FL USA; 6 Institute of Animal Science, 10-Uhrineves Prague, Czech Republic

**Keywords:** Male reproduction, Endocrine disruptor, Low dose effect, Bisphenol S, Post-translational modification

## Abstract

**Background:**

Bisphenol S (BPS) is increasingly used as a replacement for bisphenol A in the manufacture of products containing polycarbonates and epoxy resins. However, further studies of BPS exposure are needed for the assessment of health risks to humans. In this study we assessed the potential harmfulness of low-dose BPS on reproduction in male mice.

**Methods:**

To simulate human exposure under experimental conditions, 8-week-old outbred ICR male mice received 8 weeks of drinking water containing a broad range of BPS doses [0.001, 1.0, or 100 μg/kg body weight (bw)/day, BPS1–3] or vehicle control. Mice were sacrificed and testicular tissue taken for histological analysis and protein identification by nano-liquid chromatography/mass spectrometry (MS) and sperm collected for immunodetection of acetylated lysine and phosphorylated tyrosine followed by protein characterisation using matrix-assisted laser desorption ionisation time-of-flight MS (MALDI-TOF MS).

**Results:**

The results indicate that compared to vehicle, 100 μg/kg/day exposure (BPS3) leads to 1) significant histopathology in testicular tissue; and, 2) higher levels of the histone protein γH2AX, a reliable marker of DNA damage. There were fewer mature spermatozoa in the germ layer in the experimental group treated with 1 μg/kg bw (BPS2). Finally, western blot and MALDI-TOF MS studies showed significant alterations in the sperm acetylome and phosphorylome in mice treated with the lowest exposure (0.001 μg/kg/day; BPS1), although the dose is several times lower than what has been published so far.

**Conclusions:**

In summary, this range of qualitative and quantitative findings in young male mice raise the possibility that very low doses of BPS may impair mammalian reproduction through epigenetic modifications of sperm proteins.

## Introduction

Bisphenol A (BPA) is well-documented as an endocrine disruptor with detrimental effects on reproduction [1]; as a result of increasing scrutiny of BPA, there is a broad interest in substitution of alternative bisphenols for human consumption. The most common alternative bisphenol, Bisphenol S (BPS), includes a sulfone group (SO_2_) in place of the dimethylmethylene group [C (CH3)_2_] in BPA [2]. BPS has shown a range of deleterious effects following oral ingestion, inhalation or dermal absorption [3], with the most common route of intake for humans being exposure through contaminated water and food at relatively low doses [4]. To date, however, there have been only limited experimental studies of the possible harmfulness of low BPS doses.

Previous studies of BPS in male rats have reported a range of deleterious effects on hormonal balance, reduced germinal epithelium of seminiferous tubules and increased generation of reactive oxygen species [5, 6]. Recent studies have reported BPS induces epigenetic changes, including alterations in the histone code in oocytes, increased DNA methylation in mouse spermatocytes and changes to transcriptome and proteome of cells in testicular tissue and many other cells types [7–10]. Collectively, these findings suggest BPS may disrupt male reproductive functions through post-translational modifications (PTMs) of nucleic acids and proteins [1, 11, 12] and regulation of transcriptionally silenced spermatozoa [13]. In particular, lysine acetylation and tyrosine phosphorylation of sperm proteins regulate spermatogenesis and sperm capacitation [14–16]. Based on these studies, it is possible that low doses of BPS could modulate male reproduction through PTMs of protein and nucleic acid structure. BPS is classified as an endocrine disruptor and its dose-response is more likely to be nonmonotonic, hence, very-low doses may be more effective than high doses. Therefore, we have chosen wide range of much lower BPS doses than was published before [5, 6]. Using a wide range of low- and very-low doses BPS administered in drinking water for 8 weeks to young adult male mice, we want to determine the effect of BPS doses form the environment. Our findings provide one of the first indications that low doses of BPS regulate PTMs of spermatozoa and lead to possible negative effects on male reproduction.

## Material and methods

All chemicals, including BPS (CAS: 80–09-1, cat. No. 103039) were purchased from Sigma-Aldrich (USA), unless stated otherwise.

### Animals

All animal procedures were done in accordance with the Protection of Animals against Cruelty (Act No. 246/1992) under the supervision of the Animal Welfare Advisory Committee at the Ministry of Education, Youth, and Sports of the Czech Republic. Adult 7-week-old ICR male mice were purchased from Velaz Ltd. (Prague, Czech Republic), housed in standard cages in groups of 3 and maintained in a 12/12-h light/dark cycle at 21 ± 1 °C with a relative humidity of 60%. Bisphenol contamination was reduced using intact polysulfonate cages and glass drinking bottles. Mice were maintained on a phytoestrogen-free diet (1814P Altromin, Altromin Specialfutter GmbH & Co., Germany) with ultrapure water available ad libitum.

### BPS dosage and sample collection

Mice were randomized into four experimental groups and allowed to adapt for 1 week. Vehicle control (0.1% ethanol; VC) and BPS for three treatment groups were administered through drinking water at final concentrations of 0, 0.0038, 3.8, and 380 μg/L, respectively, for 8 weeks (8–16 weeks of age). The following dosages were presumed [0, 0.001, 1, and 100 μg/kg body weight (bw)/day] with actual exposure estimated based on the knowledge of recorded body weight and water intake as previously reported [17]. A wide range of doses and the route of exposure have been chosen appropriate to the real human exposure; doses of experimental animals through the drinking water have been used with respect to the welfare of animals. Hereafter, experimental groups will be stated as BPS1, BPS2 and BPS3.

Nine mice per group were included in three individual independent experiments (*n* = 36). Animal weights were recorded at the end of the experiments mice euthanised by cervical dislocation. Blood samples were collected by cardiac puncture, and serum was stored at − 80 °C until hormonal assay performance. Left and right testes were collected, weighed, and processed for histology and proteomics, respectively.

### Sperm isolation and assessment

From the mice described above, the cauda epididymidis was dissected in 0.5 mL Whitten’s medium (Suppl. Table S1), and sperm were allowed to swim out for 30 min. Thereafter, sperm concentration and motility were evaluated using Makler chamber and light microscope (Olympus CKX 41; Germany) equipped with a 10× objective (CAchN NA 0.25). 10 μl of sperm suspension was pipette to the Makler chamber, thereafter spermatozoa were counted in 3 lines, each of 10 squares and divide by 3 to obtain average sperm concentration in million per milliliter. Simultaneously, each spermatozoon across the counted area was identified either as motile or immotile. Accordingly, the sperm motility was expressed as the ratio of motile to immotile spermatozoa. The analysis was performed blindly to avoid bias.

### Hormonal profiling

Blood serum samples in three independent experiments (*n* = 5 mice per group) were assayed with Immunobeads Milliplex MAP kit (HPTP1MAG-66 K, MSHMAG-21 K; Merck Millipore, USA) for the following hormone levels: adrenocorticotropic hormone, follicle-stimulating hormone, growth hormone, luteinising hormone, thyroid-stimulating hormone, cortisol, progesterone, testosterone, triiodothyronine, and thyroxine.

### Quantitative and qualitative analyses of testes

One testis from each animal (*n* = 9 per group) was fixed in Bouin solution, embedded in paraffin wax with random orientation, and sectioned completely into 10-μm-thick slides. The total testis volume, total germ epithelium volume, and interstitium volume were estimated according to the Cavalieri principle [18]. The fractions of spermatogenesis (pre-spermiation stages I–VI; middle spermiation stages VII–VIII; post-spermiation stages IX–XII) were found using the point grid approach [19, 20]. To determine the precision and accuracy of the stereological analysis, the coefficient of error was estimated (Suppl. Tab. S2) [18]. Qualitative analysis of seminiferous tubes was performed according to the methods described by the Society of Toxicologic Pathology [21, 22] to assess the following abnormalities: missing germ cell layers and germ cell depletion, retained spermatids (spermiation failure), multinucleate and apoptotic germ cells, and exfoliation of spermatogenic cells into the lumen. At least 100 seminiferous tubules were evaluated blind to treatment group for each testicular cross section. The quantitative assessment was performed on a Nikon Eclipse Ti-U microscope (Nikon, Japan) equipped with a motorised stage (Prior, UK) using a 10× objective (Plan Fluor, NA 0.3) and Stereologer 11 software (SRC, Biosciences Tampa, FL, USA) for histopathological evaluation was performed using a 40× objective (UPlanFl, NA 0.75).

### Western blot

Testicular tissue and sperm were dissolved in lysis buffer (40 mM Tris base, 7 M urea, 2 M thiourea, 4% CHAPS, 120 mM dithiothreitol), enriched with Complete Mini Protease Inhibitor Cocktail (Roche, Switzerland), for 30 min on ice. Sperm samples of three individuals belonging to the same experimental group were pooled. Thereafter, samples were subjected to sodium dodecyl sulfate polyacrylamide gel electrophoresis on 4–15% separating Mini-PROTEAN precast gels and blotted using a Trans-Blot Turbo Transfer System onto polyvinylidene difluoride membranes (Bio-Rad Laboratories, France). The membranes were blocked in 1% bovine serum albumin in TBS with 0.5% Tween-20 for 60 min at room temperature and incubated overnight at 4 °C with primary antibodies diluted in blocking buffer. The following primary antibodies were used: anti-acetyl lysine antibodies (cat. no. ab80178; Abcam, UK), anti-phospho-tyrosine antibodies (cat. no. ab10321; Abcam), anti-acetylated α-tubulin antibodies, and anti-γH2AX antibodies. Mouse monoclonal anti-α-tubulin antibodies (cat. no. T6199; Sigma, St. Louis, MO, USA) and rabbit monoclonal anti-histone H3 antibodies (cat. no. D1H2; Cell Signaling Technology, Danvers, MA, USA) were used as the loading control for γH2AX and acetylated α-tubulin, respectively. Horseradish peroxidase-conjugated secondary antibodies (goat anti-mouse or anti-rabbit IgG; dilution: 1:15,000; Invitrogen, Carlsbad, CA, USA) were applied for 60 min at 22 °C. Target proteins were visualised using ECL Select Western Blotting Detection Reagent (GE Healthcare Life Sciences, UK) and a ChemiDoc MP System (Bio-Rad). Alternatively, proteins were visualised using a colorimetric Opti-4CN substrate kit (Bio-Rad), followed by matrix-assisted laser desorption ionisation time-of-flight (MALDI-TOF) mass spectrometry (MS) for peptide detection in the dissected bands.

### Proteome profiling

Testis lysates from animals in the experimental groups were collected for complete proteomic analysis. Nano-liquid chromatography-MS (nano-LC-MS) was used for protein identification and quantification, as described previously [7]. The acetylome and phosphorylome were analysed separately.

### Statistics

The data were processed with GraphPad Prism 8 (GraphPad Software Inc., San Diego, CA, USA). Based on Shapiro-Wilk normality distribution tests, analysis of variance (ANOVA) and Kruskal-Wallis tests were used for normally and non-normally distributed data. In cases of significant overall findings, differences between individual group pairs were assessed by Tukey’s and Dunn’s post-hoc tests, respectively. Results with *P* less than 0.05 were considered statistically significant. Normally and non-normally distributed data were expressed as means and medians, respectively.

## Results

### Hormonal profiles and sperm features of BPS-treated males

**Fig. 1:**
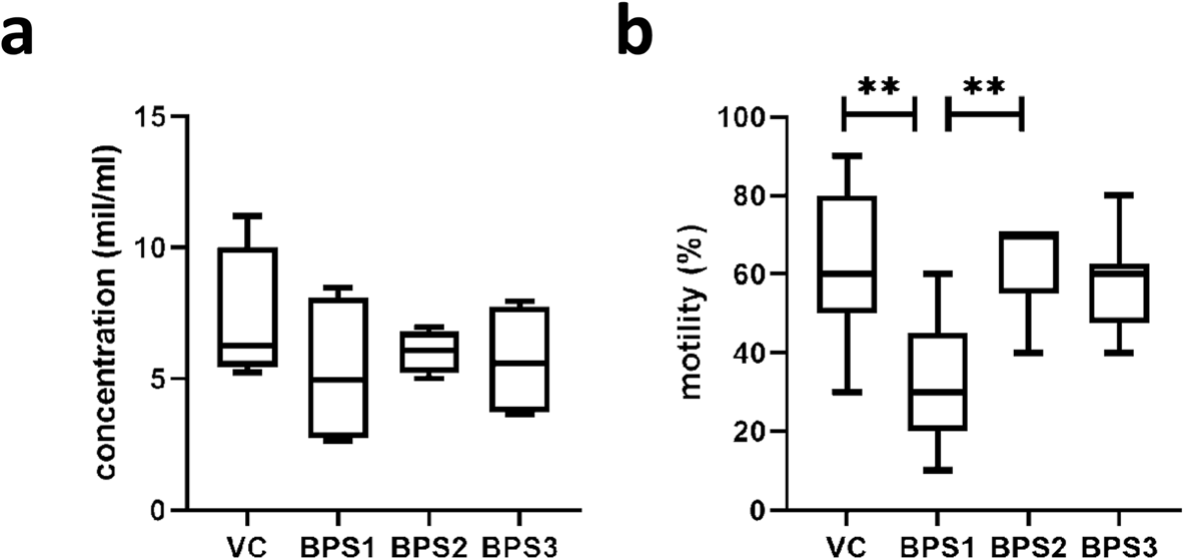
Sperm features: (**a**) sperm concentration and (**b**) percentage of motile sperm. Data are shown as medians and 5–95% percentiles. Kruskal-Wallis tests followed by Dunn’s multiple comparison tests were performed, and statistical significance is indicated (**p < 0.01). VC: vehicle control, BPS1–3: increasing doses of bisphenol S

At the end of 8-week exposure to actual doses of BPS, the body and testes weights were recorded and relative testes weights (mg/g bw) were determined. There were no differences between the experimental groups and the vehicle control (Table 1). Hormonal assays showed no significant differences in plasma hormone levels between the BPS-treated and vehicle control groups (Suppl. Table S3). Moreover, the spermatozoa count was not affected by BPS exposure (Fig. 1a), although treatment with 0.001 μg/kg bw BPS1 decreased the portion of motile spermatozoa (Fig. 1b).

**Table 1:** Characteristics of experimental animals

	VC	BPS1	BPS2	BPS3
Weight of mouse body (g)	41,82 ± 0,72^a,b^	42,44 ± 0,77^a,b^	45,24 ± 1,36^a^	41,31 ± 1^b^
Relative weight of testes (mg/g of bw)	12,81 ± 0,30	12,76 ± 0,15	10,87 ± 0,37	11,03 ± 0,17

Body and relative testis weights are shown as means ± SEM of animals included in the study (*n* = 9 per experimental group). One-way ANOVA was followed by Tukey’s multiple comparison tests. Different letters in the same row indicate significant differences (*p* < 0.05). *VC* vehicle control, *BPS1–3* increasing doses of bisphenol S

### Higher BPS exposure induced abnormal testicular histopathology

**Fig. 2:**
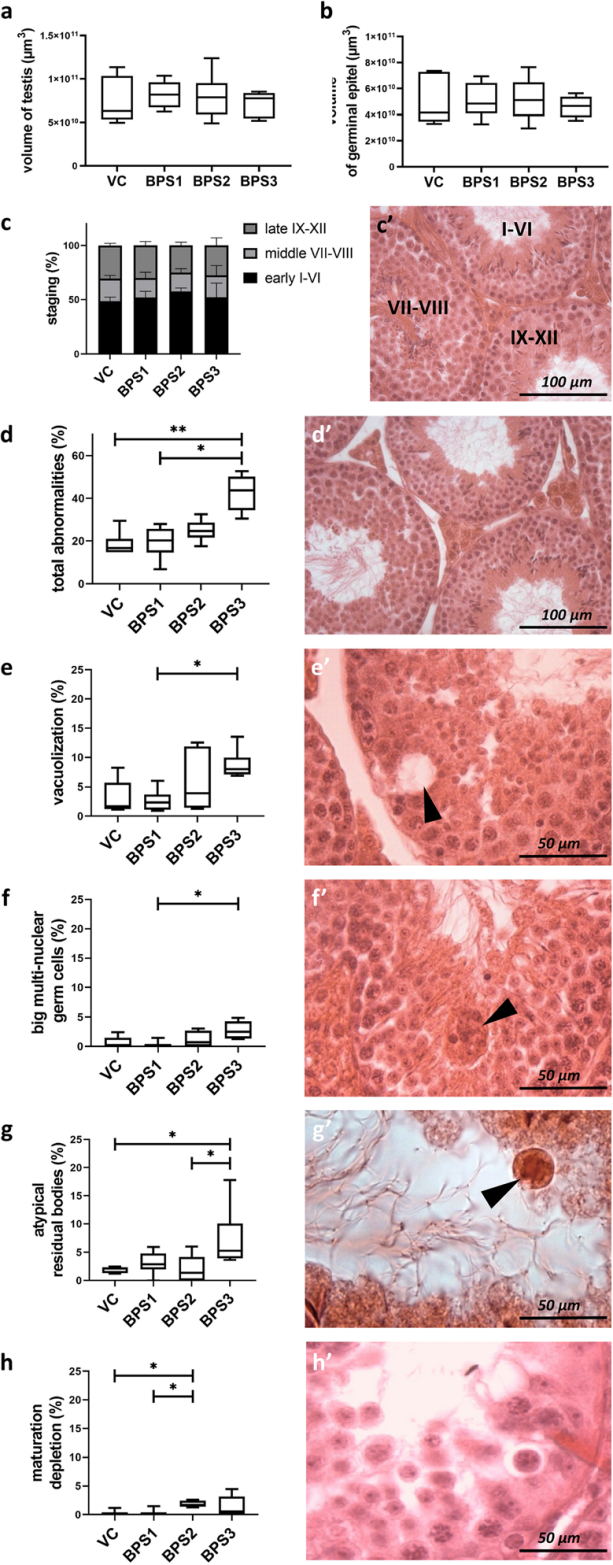
Stereological and histopathological analyses of mouse testis in different treatment groups. (**a**) Fluctuations in the total testis volume (μm^3^) in experimental groups; (**b**) volume of germinal epithelium (μm^3^); and (**c, c’**) stage of spermatogenesis (%) were recorded. Histopathological manifestations were tracked as follows: (**d**) portion of seminiferous tubule profiles containing an abnormality (%), including (**e**) tubes with vacuolisation (%), (**f**) tubes carrying large multinuclear germ cells (%), (**g**) atypical residual bodies (%), and/or (**h**) maturation depletion (%). (d’) Representative images of healthy germ epithelium and (**e’–h’**) individual abnormalities, respectively, are shown and indicated with arrowheads. Data are expressed as medians and 5–95% percentiles of six animals per experimental group. Kruskal-Wallis tests, followed by Dunn’s multiple comparison tests, were performed, and statistical significance is indicated (*p < 0.05, **p < 0.01). VC: vehicle control, BPS1–3: increasing doses of bisphenol S

Histological assessment was performed to evaluate the impact of actual BPS doses on testicular tissues. Stereological analysis showed no differences between groups in terms of testis volume, germinal epithelium volume (Fig. 2a, b), interstitium volume, germ layer volume fraction, and interstitium volume fraction. To investigate the effects of BPS treatments on spermatogenesis, individual stages of the seminiferous epithelium were identified and no differences between experimental groups were found (Fig. 2c, c’). Histopathological analysis of testicular tissues from BPS-exposed male mice showed an increased incidence of abnormalities in mice treated with the highest BPS dose (BPS3; Fig. 2d). In addition to vacuolisation of germ layer cells and enlarged multi-nuclear germ cells, the atypical residual bodies demonstrated the effects of BPS3 on testicular tissues (Fig. 2d–g). There were fewer mature spermatozoa in the germ layer in the BPS2 experimental group (Fig. 2h). Representative images of individual histopathologies are shown (Fig. 2d’–h’).

### Proteomic analysis of testicular tissue

**Fig. 3:**
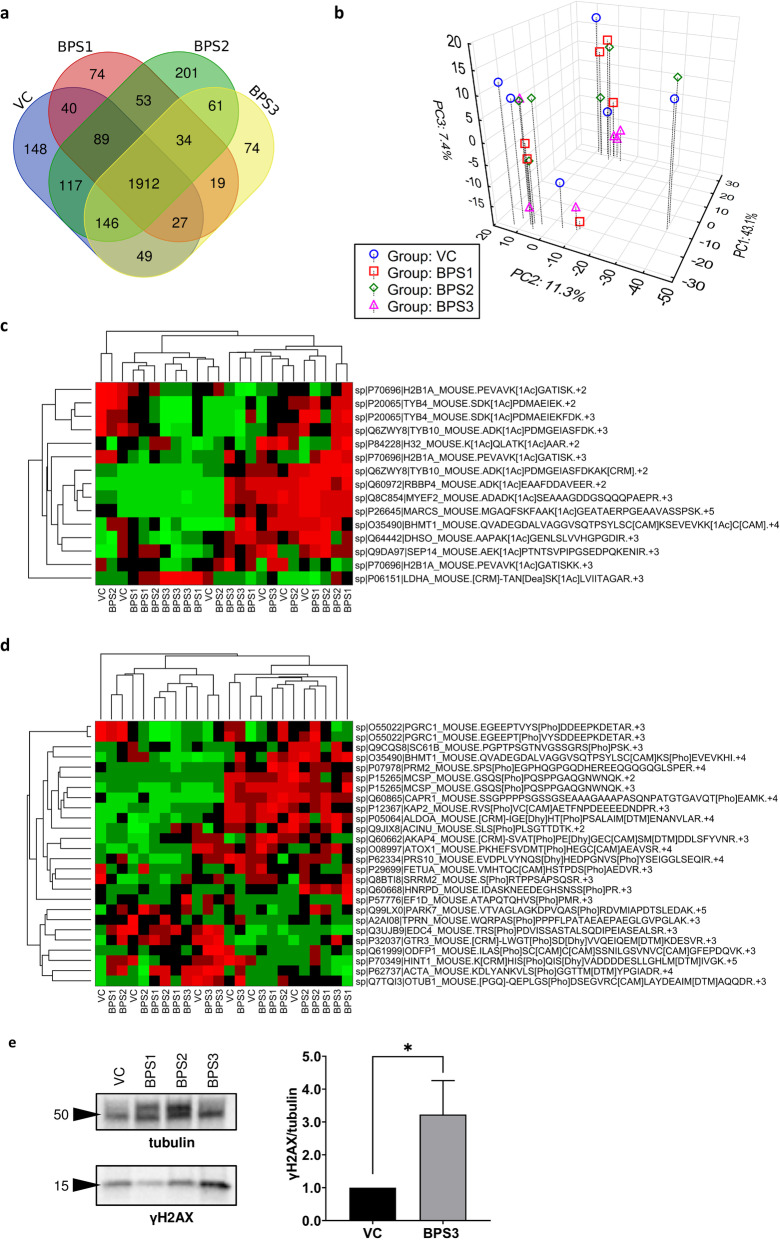
Proteomic analysis of testicular tissues. (**a**) Venn diagram of total described testicular proteins in mice (*n* = 4) after various treatments in different experimental groups. (**b**) Projection of 24 experimental mice into the space of first three principal components according to PCA; percentages in the axis legends show the proportion of total variance explained by the particular component. (**c**) Overview of acetylated and (**d**) phosphorylated testicular proteins. (**e**) Analysis of γH2AX; band signals were normalized to α-tubulin and related to the vehicle control as the mean (range) of three independent experiments. Unpaired t tests were performed, and statistical significance is indicated (*p < 0.05). VC: vehicle control, BPS1–3: increasing doses of bisphenol S

Based on the different modes of action of BPS at various doses, whole-proteome profiling of testicular tissues was performed. In total, 3044 proteins were detected. Unique protein expression in the control and BPS-treated groups is shown in the Venn diagram in Fig. 3a. However, after quantification of the levels of 1886 proteins, followed by subsequent principle component analysis (PCA), no distinct clusters of mice (*n* = 24) from individual groups were observed, thus indicating a lack of a consistent proteome pattern (Fig. 3b). In addition to total protein analysis, acetylated (*n* = 15) and phosphorylated (*n* = 26) peptides were quantified (Fig. 3c, d), and no significant differences were observed. Moreover, the deleterious effects of BPS3 were elucidated using antibodies against the phosphorylated form of H2AX (γH2AX) to label DNA double-stranded breaks; γH2AX is a representative PTM that can be used to identify DNA damage and cellular stress. Consistent with the increased incidence of abnormalities in seminiferous tubules in the BPS3 group, we observed an increase in the γH2AX signal as well (Fig. 3e).

### Lower BPS exposure changed the post-translational quality of sperm proteins

**Fig. 4:**
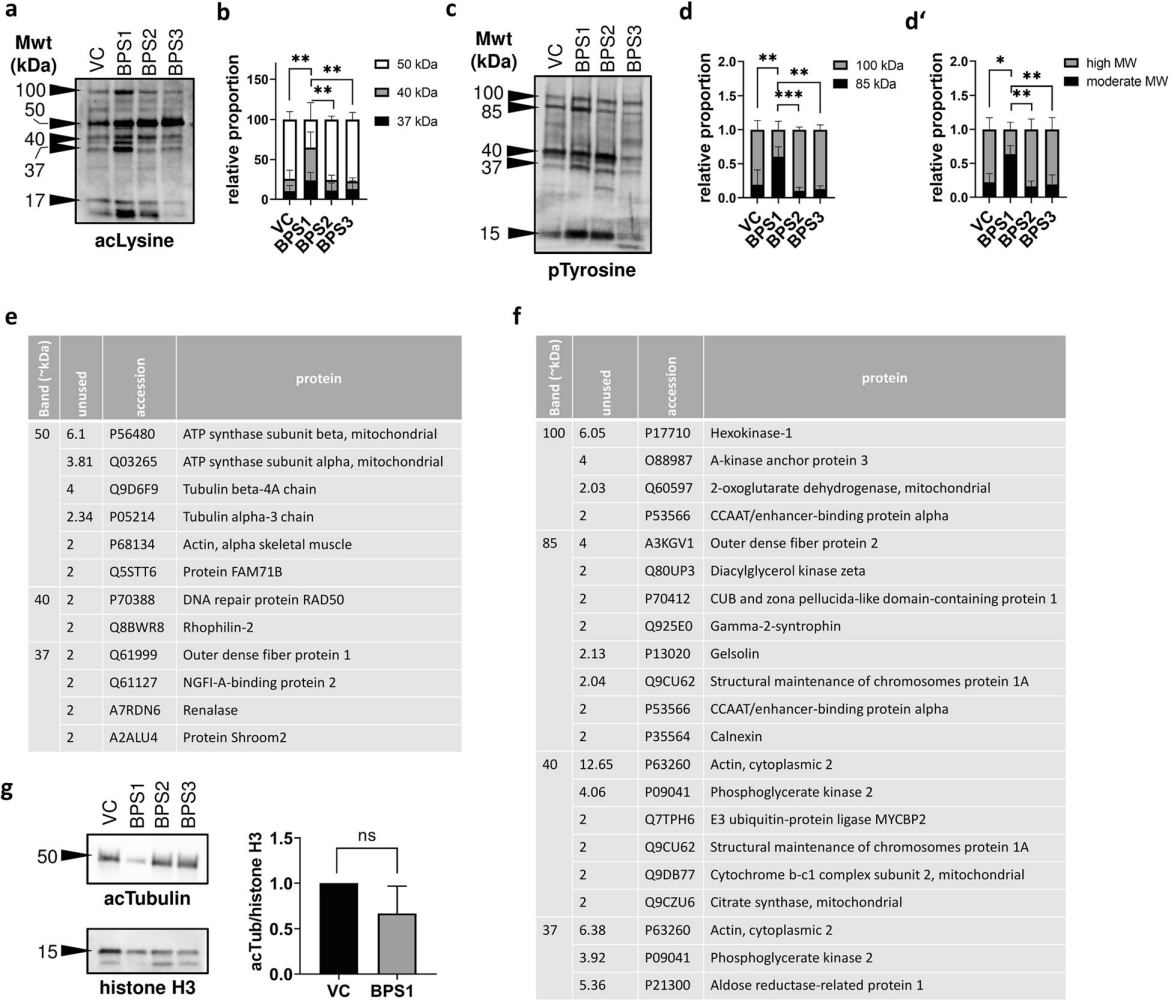
Sperm acetylome and phosphorylome analyses. (**a**) Acetylated sperm proteins (acetylated lysine) with major bands. (**b**) Densitometric analysis of the ratio of candidate bands. (**c**) Phosphorylated sperm proteins (phosphorylated tyrosine) with major bands. (**d**) Densitometric analysis of the ratio of 100- to 85-kDa bands. (d’) The ratio of 37–40-kDa (moderate) to 85–100-kDa (high) molecular weight bands. Differential counting was expressed as means (ranges) of three independent experiments. Differences were tested by two-way ANOVA, followed by Tukey’s multiple comparison test, and asterisks indicate statistical significance *p < 0.05, **p < 0.01, ***p < 0.001, and ****p < 0.0001. (**e**) Candidate acetylated and (**f**) phosphorylated proteins from individual bands were evaluated using MALDI-TOF MS-based peptide detection. Analysed sperm samples represent a pool of three animals per experimental group from three independent replicates. (**g**) Densitometric analysis of acetylated tubulin from BPS1-treated sperm. VC: vehicle control, BPS1–3: increasing doses of bisphenol S

In accordance with whole-proteome analysis of BPS-treated testes, analyses of the sperm acetylome and phosphorylome were performed using western blotting and MALDI-TOF MS. Because of the low protein amounts in sperm lysate extracts, sperm samples from three individuals belonging to the same experimental group were pooled, and three independent experiments were performed. After loading equal amounts of protein per well, we found that the acetylation of proteins with molecular weights of approximately 37, 40, and 50 kDa were affected by treatment with BPS1 (Fig. 4a, b). Moreover, BPS1 also modified the phosphorylation of sperm proteins (37, 40, 85, and 100 kDa; Fig. 4c, d, d’). Candidate acetylated and phosphorylated proteins are summarised in Fig. 4 (e, f), and the results indicated the involvement of housekeeping proteins (ATP synthase subunit, hexokinase-1) and enzymes (DNA repair protein, E3 ubiquitin-protein ligase). In accordance with previous findings, demonstrating that BPS1 suppressed sperm motility, cytoskeletal factors (i.e., tubulin chains, actin; Fig. 4e) seems to be underwent to acetylation. Therefore, an antibody against acetylated α-tubulin (acTubulin) was used for a verification of tubulin as a candidate BPS target.

Next, we evaluated the densitometry of bands representing acetylated tubulin after treatment with BPS1 (Fig. 4g). Decreased tubulin acetylation was observed; however, the difference was not statistically significant, suggesting that other targets (such as ATP synthase and actin) may be related to sperm motility.

## Discussion

Male reproduction involves sensitive machinery, which is required for spermatozoon development and can be affected by exposure to various environmental stimuli. Because mature spermatozoa have been transcriptionally silenced, changes in PTMs can regulate protein activity and modify other crucial biomolecules. Indeed, lysine acetylation and phosphorylation have been shown to be indispensable for the proper function of sperm [14, 15]. Our findings suggested that PTMs may be affected by pollutants from the environment. In our study, we simulated the exposure of adult males to BPS, a common endocrine disruptor, at very low doses (~ 0.001 and 1 μg/kg bw/day). Moreover, we chose ~ 100 μg/kg bw, which has been suggested to induce reproductive toxicity [3, 5]. The 8-week exposure covered the whole duration of spermatogenesis; therefore, we assumed that the sperm quality and testicular tissues would be affected at the tissue/cell and proteome levels. We also evaluated the effects of endocrine disruption on post-translational modifications of testicular/sperm proteins in accordance with our hypothesis of the “post-translational effect” of very low doses of these agents.

Recent studies have demonstrated that bisphenols alter steroid signalling pathways, having negative effects on male and female reproduction. Our observations did not reveal hormonal changes, even after higher BPS exposure, whereas comparable doses were found to be effective in rats [6]. However, earlier results showing that endocrine disruptors lead to hormonal imbalances should be revised because alternative mechanisms of hormone-derived actions have been noted. For example, oestrogen-like action results in carcinogenesis [23], and changes in the distributions of oestrogen receptors and androgen-converting enzyme aromatase have been reported [24]. Endocrine disruptors have also been shown to modulate downstream signalling of activated G protein-coupled oestrogen receptors [25]. It is difficult to identify bisphenol-affected mechanisms after systemic exposure; therefore, cellular and molecular markers are appropriate for assessment of the risk of bisphenol exposure. Based on our findings, we speculate that different doses of BPS may have different effects. For example, whereas extremely low doses (BPS1: ~ 0.001 μg BPS/kg bw) affected sperm motility, higher BPS doses (BPS3: ~ 100 μg/kg bw) showed significant effects on testicular tissues. Surprisingly, moderate doses of BPS (BPS2: equal to daily intake of approx. 0.1 μg/kg bw) did not show any effects on spermiogram recording and histological assessment. This finding was consistent with the phenomenon of nonlinear effects [26], with the lowest dose of BPS (BPS1) inducing motility failure rather compared with the other BPS doses. Therefore, proteome profiling was used to test a wide range of BPS doses and characterise the dose-dependent mode of action.

Because of the lack of effect of BPS on the whole proteome of testicular tissues, protein acetylation and phosphorylation were chosen for further analysis. Although no significant effects were observed in terms of acetylation and phosphorylation of the detected peptides, γH2AX, a mark of DNA damage, was increased in BPS3 testicular tissues, demonstrating the increased occurrence of abnormalities. In sperm lysates, protein acetylation and phosphorylation were detected using specific antibodies against acetylated lysine and phosphorylated tyrosine. The choice of PTMs was consistent with the earlier described biological role of both PTMs in sperm capacitation and fertilisation ability [14, 27]. Indeed, altered levels of acetylated and phosphorylated proteins were observed after exposure to very-low-dose BPS (BPS1). This finding was presumably associated with decreased motility, resulting in detection of candidate proteins. We can assume that differentially acetylated and/or phosphorylated may be responsible for motility failure, in accordance with the significance of PTMs in major proteins, including phospho-hexokinase-1 [28] and phospho-outer dense fibre protein-2 [29]. Decreased phospho-tyrosine signals at 100 kDa suggest a lack of hexokinase-1 activity, which is associated with male infertility [30]. Our findings supported the mechanism of action of BPA described previously through fertility-related proteins, including protein phosphorylation [31]. Our study suggested that in addition to phosphorylation, bisphenols altered other PTMs, particularly protein acetylation. However, western blot analysis using anti-acetylated tubulin did not show any decrease, as expected, and other protein targets for acetylation were considered, including ATP synthase and actin, both of which are involved in sperm motility [32, 33].

## Conclusion

In conclusion, these studies are among the first to raise the possibility that low and very low doses of BPS may have a deleterious effect on sperm quality in mammals. Since human BPS exposure is much lower (0.004 μg/kg bw/day) than is commonly tested [34], our findings suggest that post-translational effects could play a role in idiopathic infertility. Furthermore, this work supports the view that substitution of BPS for BPA may be inadequate for elimination of the negative effects of these agents on public health. Further biomonitoring and testing of molecular targets of BPS could be relevant for accurate risk assessment and elimination of its potential negative impact on male fertility.

## Supplementary information

**Additional file 1: Table S1.** Composition of Whitten's HEPES-buffered medium. **Table S2.** Coefficients of error (CE) for evaluated terms of performed stereological analysis (*n* = 9 per each group). VC: vehicle control, BPS1-3: increasing doses of bisphenol S. **Table S3.** Hormone profiling of males in different experimental groups. Values of adrenocorticotropic hormone (ACTH), follicle-stimulating hormone (FSH), growth hormone (GH), luteinising hormone (LH), thyroid-stimulating hormone (TSH), cortisol, progesterone, testosterone, triiodothyronine (T3), and thyroxine (T4) are expressed as medians ± SEM, n = 5 per experimental group. Kruskal-Wallis tests were followed by Dunn’s multiple comparison tests. Different letters in the same row indicate significant differences (*p* < 0,05). VC: vehicle control, BPS1-3: increasing doses of bisphenol S.

## Data Availability

The datasets used and/or analyzed during the current study are available from the corresponding author on reasonable request.

## Abbreviations

BPA: Bisphenol A
BPS: Bisphenol S
CHAPS: 3-[(3-Cholamidopropyl) dimethylammonio]-1-propanesulfonate hydrate
MALDI-TOF MS: Matrix-assisted laser desorption ionisation time-of-flight mass spectrometry
MS: Mass spectrometry
nano-LC-MS: Nano-liquid chromatography mass spectrometry
PTMs: Post-translational modifications
TBS: Tris-buffered saline
VC: Vehicle control
