# Supplementary material for: Low doses of Bisphenol S affect post-translational modifications of sperm proteins in male mice
Source: Reprod Biol Endocrinol. 2020 May 28;18:56. doi: 10.1186/s12958-020-00596-x (PMC7254721; doi:10.1186/s12958-020-00596-x)
Supplement: Supplementary file 1 — Additional file 1: Table S1. Composition of Whitten's HEPES-buffered medium. Table S2. Coefficients of error (CE) for evaluated terms of performed stereological analysis (n = 9 per each group). VC: vehicle control, BPS1-3: increasing doses of bisphenol S. Table S3. Hormone profiling of males in different experimental groups. Values of adrenocorticotropic hormone (ACTH), follicle-stimulating hormone (FSH), growth hormone (GH), luteinising hormone (LH), thyroid-stimulating hormone (TSH), cortisol, progesterone, testosterone, triiodothyronine (T3), and thyroxine (T4) are expressed as medians ± SEM, n = 5 per experimental group. Kruskal-Wallis tests were followed by Dunn’s multiple comparison tests. Different letters in the same row indicate significant differences (p < 0,05). VC: vehicle control, BPS1-3: increasing doses of bisphenol S. [file 12958_2020_596_MOESM1_ESM.docx]

**Supplementary table S1.** Composition of Whitten's HEPES-buffered medium

| Reagent | Molarity (mM) |
| --- | --- |
| NaCl (Sigma Aldrich, S-7653) | 100,00 |
| KCl (Sigma Aldrich, P-5405) | 4,40 |
| KH_2_PO_4_ (Sigma Aldrich, P-5655) | 1,20 |
| MgSO_4_ , 7 H_2_O (Sigma Aldrich, 63140) | 1,20 |
| Glucose (Sigma Aldrich, G-6152) | 5,40 |
| Calcium L,lactate (hydrate) (Sigma Aldrich, L-4388) | 4,80 |
| HEPES (Sigma Aldrich, H-7523) | 20,00 |
| Sodium Pyruvate (Sigma Aldrich, P-4562) | 0,8 |

pH was adjusted at 7,2 – 7,4

**Supplementary Table S2.** Coefficients of error (CE) for evaluated terms of performed stereological analysis (*n* = 9 per each group). VC: vehicle control, BPS1-3: increasing doses of bisphenol S.

|  | VC | BPS1 | BPS2 | BPS3 |
| --- | --- | --- | --- | --- |
| CE for Volume of testes | 0,012 | 0,011 | 0,011 | 0,012 |
| CE for volume of germ epithelium | 0,073 | 0,074 | 0,071 | 0,072 |
| CE for volume of intersticium | 0,129 | 0,119 | 0,122 | 0,122 |

**Supplementary table S3.** Hormone profiling of males in different experimental groups.

Values of adrenocorticotropic hormone (ACTH), follicle-stimulating hormone (FSH), growth hormone (GH), luteinising hormone (LH), thyroid-stimulating hormone (TSH), cortisol, progesterone, testosterone, triiodothyronine (T3), and thyroxine (T4) are expressed as medians ± SEM, *n* = 5 per experimental group. Kruskal-Wallis tests were followed by Dunn’s multiple comparison tests. Different letters in the same row indicate significant differences (*p* < 0,05). VC: vehicle control, BPS1-3: increasing doses of bisphenol S.

|  | VC | BPS 1 | BPS 2 | BPS 3 |
| --- | --- | --- | --- | --- |
| ACTH | 1,43 ± 0,61 | 1,07 ± 0,38 | 0,63 ± 1,73 | 1,87 ± 1,07 |
| FSH (mIU/ml) | 61,67 ± 31,84 | 45,69 ± 39,17 | 58,11 ± 11,94 | 86,81 ± 26,69 |
| GH (pg/ml) | 0,13 ± 0,18^a,b^ | 0,85 ± 0,24^a,b^ | 1,43 ± 0,65^a^ | 0 ± 0,00^b^ |
| LH (mIU/ml) | 2,22 ± 3,13 | 0,18 ± 0,05 | 0,58 ± 0,55 | 0,4 ± 0,21 |
| TSH (µIU/ml) | 0,03 ± 0,03 | 0,01 ± 0,01 | 0,01 ± 0,05 | 0,01 ± 0,09 |
| Cortisol (ng/ml) | 9,31 ± 1,55 | 6,47 ± 1,56 | 0,88 ± 1,30 | 2,06 ± 2,05 |
| Progesterone (ng/ml) | 0,78 ± 0,33 | 1,33 ± 0,20 | 0,70 ± 0,24 | 0,67 ± 0,69 |
| Testosterone (ng/ml) | 9,61 ± 5,02^a,b^ | 17,26 ± 3,92^a,b^ | 27,91 ± 5,84^a^ | 1, ± 1,77^b^ |
| T3 (ng/ml) | 1,87 ± 0,19 | 1,93 ± 0,12 | 1,87 ± 0,40 | 2 ± 0,14 |
| T4 (ng/ml) | 64,59 ± 2,23 | 65,07 ± 1,97 | 64,16 ± 3,38 | 45,49 ± 5,18 |
